# A Novel Missense Variant of *TP63* Heterozygously Present in Split-Hand/Foot Malformation

**DOI:** 10.1155/2020/4215632

**Published:** 2020-11-26

**Authors:** Hao Geng, Dongdong Tang, Chuan Xu, Xiaojin He, Zhiguo Zhang

**Affiliations:** ^1^Reproductive Medicine Center, Department of Obstetrics and Gynecology, The First Affiliated Hospital of Anhui Medical University, No. 218 Jixi Road, Hefei, 230022 Anhui, China; ^2^NHC Key Laboratory of Study on Abnormal Gametes and Reproductive Tract (Anhui Medical University), No. 81 Meishan Road, Hefei, 230032 Anhui, China; ^3^Key Laboratory of Population Health across Life Cycle (Anhui Medical University), Ministry of Education of the People's Republic of China, No. 81 Meishan Road, Hefei, 230032 Anhui, China

## Abstract

**Background:**

Split-hand/foot malformation (SHFM) is a severe congenital disability mainly characterized by the absence or hypoplasia of the central ray of the hand/foot. To date, several candidate genes associated with SHFM have been identified, including *TP63*, *DLX5*, *DLX6*, *FGFR1*, and *WNT10B*. Herein, we report a novel variant of *TP63* heterozygously present in affected members of a family with SHFM.

**Methods:**

This study investigated a Chinese family, in which the proband and his son suffered from SHFM. The peripheral blood sample of the proband was used to perform whole-exome sequencing (WES) to explore the possible genetic causes of this disease. Postsequencing bioinformatic analyses and Sanger sequencing were conducted to verify the identified variants and parental origins on all family members in the pedigree.

**Results:**

By postsequencing bioinformatic analyses and Sanger sequencing, we identified a novel missense variant (NM_003722.4:c.948G>A; p.Met316Ile) of *TP63* in this family that results in a substitution of methionine with isoleucine, which is probably associated with the occurrence of SHFM.

**Conclusion:**

A novel missense variant (NM_003722.4:c.948G>A; p.Met316Ile) of *TP63* in SHFM was thus identified, which may enlarge the spectrum of known *TP63* variants and also provide new approaches for genetic counselling of families with SHFM.

## 1. Introduction

Split-hand/foot malformation (SHFM) is a severe congenital abnormality mainly characterized by the absence or hypoplasia of the central rays of the hand/foot, which can be isolated or syndromic [1]. The reported incidence of SHFM ranges from 1/6000 to 1/20000, worldwide. The incidence in China could be higher, underlying higher disabilities in infants [2, 3]. Genetic and environmental factors have been proven to contribute significantly to the occurrence of congenital malformations. Several candidate genes have been reported to be associated with SHFM, including *TP63* (OMIM 603273), *DLX5* (OMIM 600028), *DLX6* (OMIM 600030), *FGFR1* (OMIM 136350), *WNT10B* (OMIM 601906), and *BHLHA9* (OMIM 615416). The majority of SHFM cases display autosomal dominant inheritance, but other modes of inheritance have also been described [4, 5]. In addition, environmental exposure to medication and chemicals also increases the risk of limb malformations [6, 7].

In the present study, we investigated an isolated Chinese family with no history of exposure to environmental risk factors. In this family, the proband and his son suffered from SHFM. Whole-exome sequencing (WES) was used to detect possible genetic lesions, and a novel missense variant (NM_003722.4:c.948G>A; p.Met316Ile) of *TP63* was identified to be associated with the occurrence of SHFM in this family.

## 2. Materials and Methods

### 2.1. Subjects

We investigated 3 generations of a Chinese family from Anhui province, with four family members participating in our study. The proband and his son suffered from SHFM. Peripheral blood samples were collected from all family members for genetic analyses. Clinical symptoms and imaging results of the affected individuals were also recorded. All participants signed informed consent, and this study was approved by the local ethics committee.

### 2.2. WES, Postsequencing Bioinformatic Analyses, and Sanger Sequencing

Genomic DNA was extracted from peripheral blood samples taken from all participants using a DNA blood mini kit (Qiagen, Germany). After quality control, the proband's DNA was used for WES, performed by the Beijing Genomics Institute (Shenzhen, China) with a MGISEQ-2000 genetic sequencer. Procedures were described as follows: (1) library prepared and assessed, (2) sequenced by MGISEQ-2000, (3) reads aligned with hg19 using BWM and GATK software after data filtering, (4) variants identified and annotated (1KGP, ExAC_all, gnomAD, OMIM, ClinVar, HGMD, SIFT, PolyPhen-2, and MutationTaster), and (5) variants validated by Sanger sequencing and cosegregation analysis. Detailed method information has been provided in a previous study [8].

## 3. Results

**Figure 1:**
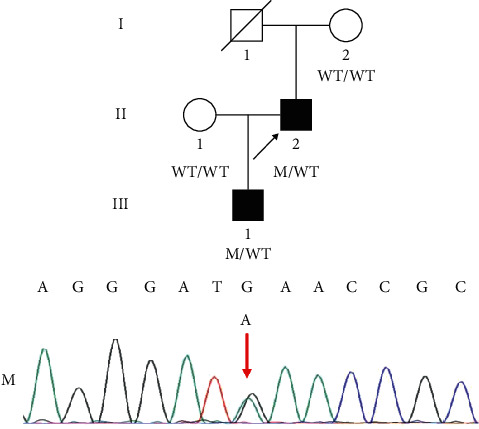
Variant of *TP63* identified in a Chinese family with SHFM (NM_3722.4:c.948G>A). The proband (II-2) and his son (III-1) were heterozygous for this variant. The red arrow indicates variant information in Sanger sequencing. Abbreviations: SHFM = split-hand/foot malformation; WT = wild type; M = *TP63* variant.

**Figure 2:**
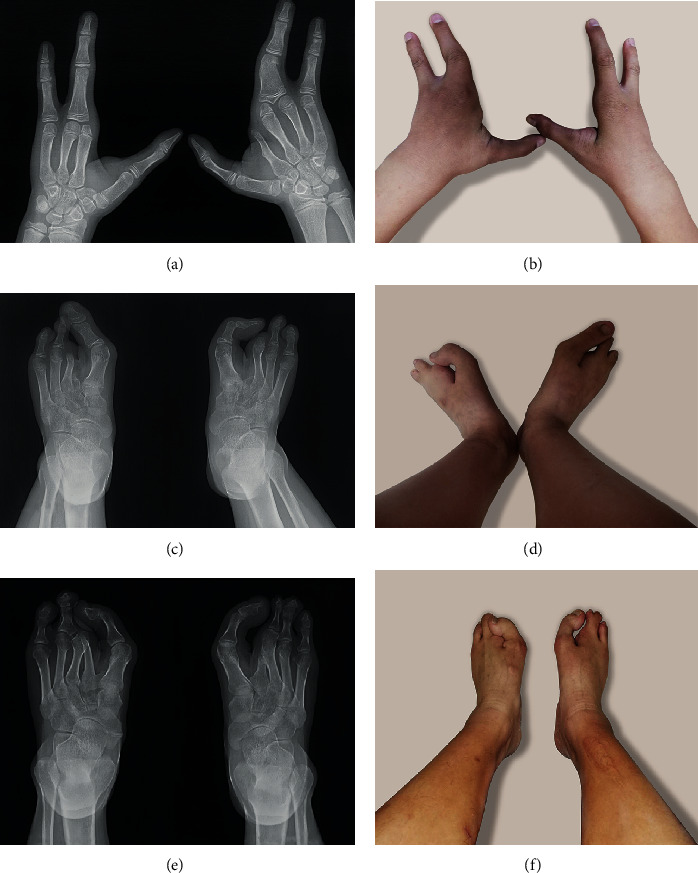
(a–d) Clinical phenotypes and X-rays of the proband's son (III-1). (e, f) Clinical phenotypes and X-rays of the proband (II-2).

This study identified a family with two members (II-2 and III-1) diagnosed with SHFM (Figure 1). The proband (II-2), who already had a child with SHFM, went to the reproductive centre for fertility counselling. The proband experienced bilateral split-foot malformations, and his son suffered from cleft hand and foot deformities. No other abnormities were found in the proband or his son. The clinical and imaging features of the affected individuals are shown in Figure 2. Notably, in this family, the proband's father (I-1) died before seeking genetic counselling; thus, the clinical features were not recorded. However, based on descriptions given by his family members, he did not show any clinical signs of limb malformations.

**Figure 3:**
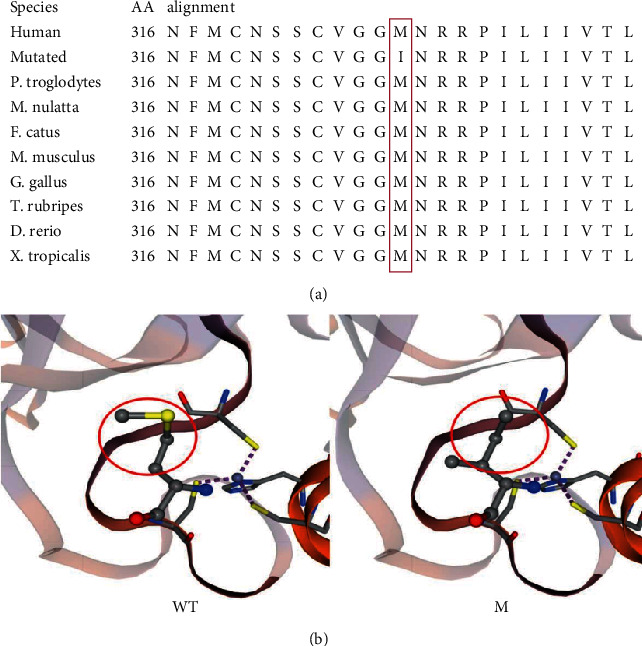
(a) The novel variant (p.Met316Ile) is located in the highly conserved site among species. The red letter represents the mutated amino acid; (b) the partial structure of TP63 protein constructed by Swiss-model with red circles emphasizing the changed conformation. WT = wild type. M = *TP63* variant.

Using WES, we identified a novel heterozygous variant (NM_003722.4:c.948G>A; p.Met316Ile) of *TP63* in the proband and his son (Figure 1). This new variant is not found in the gnomAD, 1000G, and ExAC databases (Table 1). An amino acid sequence alignment suggests that the 316th amino acid in TP63 protein is highly conserved among different species (Figure 3(a)). This novel variant was predicted to be disease-causing/probably damaging by MutationTaster and PolyPhen-2 (Table 1). Subsequently, we constructed a partial model of TP63 protein using Swiss-model; the mutated one exhibits an altered three-dimensional structure of TP63 (Figure 3(b)). Finally, Sanger sequencing found this new variant in affected family members but not in healthy individuals, conforming to the cosegregation principle.

## 4. Discussion

SHFM is a severe congenital heterogeneous limb abnormality that mainly affects the development of the central rays in the hand/foot. It may occur in an isolated or syndromic manner. The clinical phenotypes of SHFM are highly variable, ranging from hypoplasia in a single phalanx or syndactyly to aplasia in one or more central limbs [9]. The development of limbs is a very complex process that begins with the formation of limb buds. The apical ectodermal ridge (AER), located at the distal edge of the developing limb bud, acts as the main signal centre regulating growth along the proximal/distal axis. Disruption of the AER may contribute to SHFM [4].

Recently, it has been reported that genetic factors play a crucial role in the occurrence of SHFM. Several chromosomal loci have been identified that associate with the occurrence of SHFM. Chromosomal rearrangements in 7q21 lead to SHFM1; *DLX5* and *DLX6* located in this area are involved in the development of limb malformation [10, 11]. SHFM2 is caused by mutations in Xq26 [12]. Duplications involving *BTRC* and *FBXW4* in 10q24 contribute to the occurrence of SHFM3 [13, 14]. SHFM4-associated mutations mapping to 3q28 have been found to be in *TP63* [15–17]. Dysregulation of the *HOXD* gene cluster located in 2q31 plays a key role in SHFM5 [18]. *WNT10B* mutations in 12q13 are involved in the development of SHFM6 [19, 20]. In addition, there exists a specific SHFM with tibia and fibula deficiency called SHFMD. *BHLHA9*-associated duplications in 17p13 display significant association with SHFMD [21]. SHFM1, 3, 4, and 5 mainly exhibit an autosomal dominant inheritance pattern, while SHFM2 and 6 display X-linked and autosomal recessive models of inheritance, respectively.

Heterozygous expression of mutant TP63 could underlie the occurrence of SHFM4 [4, 5]. Hence, it is essential to provide families with histories of SHFM with molecular genetic testing and counselling. In the present study, we identified a novel heterozygous variant of *TP63* in an isolated SHFM family. Based on clinical features and WES results, this type was diagnosed as SHFM4, probably inherited in an autosomal dominant inheritance pattern. However, the proband's father died before molecular testing; although he did not show any clinical signs of limb malformations, we cannot exclude paternal inheritance.

*TP63* is a protein-coding gene comprising 17 exons, 2 promoters, and some variable splice sites. The TP63 isoforms encoded by this gene can be divided into two categories (TAp63 and *Δ*Np63) whose expression is driven by different promoters. TAp63 isoforms own an N-terminal transactivation (TA) domain, which is absent in *Δ*Np63 isoforms. Both the TAp63 and *Δ*Np63 isoforms can be further divided into TAp63 and *Δ*Np63*α*, *β*, and *γ* variants after undergoing mRNA alternative splicing. TAp63*α* is the longest isoform, containing a TA domain, a central DNA-binding domain (DBD), an oligomerization domain (OD), a C-terminal Sterile Alpha Motif (SAM), and a Transactivation Inhibitory (TI) domain [22–25].

As a member of the p53 family of transcription factors, TP63 plays a key role in the formation and differentiation of the AER and is crucial to limb development [4]. The newly discovered amino acid substitution (p.Met316Ile) confirmed in this study occurred at a mutational hotspot in DBD, which is responsible for DNA binding. According to the Alamut Visual software and the ACMG 2015 guidelines, this variant is regarded as a class 3-unknown pathogenicity. However, this site in TP63 is evolutionarily highly conserved among different species. Despite there was small physicochemical difference between Met and Ile according to Grantham scores, bioinformatics software (MutationTaster and PolyPhen-2) predicted that this new variant would be disease-causing/probably damaging. Importantly, Swiss-model software also suggested that this novel variant may change the TP63 partial structure in its DNA-binding domain, which may affect the formation and differentiation of the AER, probably leading to limb malformation.

In conclusion, a novel heterozygous missense variant (NM_003722.4:c.948G>A; p.Met316Ile) of *TP63* was detected in a Chinese family by whole-exome sequencing. It must be included in genetic diagnoses and counselling discussions of families with SHFM.

## Figures and Tables

**Table 1:** *TP63* variant (NM_003722.4:c.948G>A; p.Met316Ile) in a Chinese family with SHFM.

Gene	*TP63*
DNA change	NM_003722.4:c.948G>A (heterozygous)
Amino acid alteration	p.Met316Ile
Variant type	Missense
*Allele frequency*	
1KGP	0
ExAC_all	0
gnomAD	0
*Function prediction*	
MutationTaster	Disease causing (1.000)
PolyPhen-2	Probably damaging (0.937)
SIFT	Tolerated (0.074)

Abbreviations: SHFM: split-hand/foot malformation; 1KGP: 1000 Genomes Project; ExAC_all: all the data of Exome Aggregation Consortium; gnomAD: the Genome Aggregation Database.

## Data Availability

The datasets used and/or analyzed during the current study are available from the corresponding authors on reasonable request.
